# Epigenetic regulation of nuclear processes in fungal plant pathogens

**DOI:** 10.1371/journal.ppat.1011525

**Published:** 2023-08-03

**Authors:** H. Martin Kramer, David E. Cook, Michael F. Seidl, Bart P.H.J. Thomma

**Affiliations:** 1 Laboratory of Phytopathology, Wageningen University and Research, Wageningen, the Netherlands; 2 Department of Plant Pathology, Kansas State University, Manhattan, Kansas, United States of America; 3 Theoretical Biology & Bioinformatics, Department of Biology, Utrecht University, Utrecht, the Netherlands; 4 University of Cologne, Institute for Plant Sciences, Cluster of Excellence on Plant Sciences (CEPLAS), Cologne, Germany; University of Exeter, UNITED KINGDOM

## Abstract

Through the association of protein complexes to DNA, the eukaryotic nuclear genome is broadly organized into open euchromatin that is accessible for enzymes acting on DNA and condensed heterochromatin that is inaccessible. Chemical and physical alterations to chromatin may impact its organization and functionality and are therefore important regulators of nuclear processes. Studies in various fungal plant pathogens have uncovered an association between chromatin organization and expression of in planta*-*induced genes that are important for pathogenicity. This review discusses chromatin-based regulation mechanisms as determined in the fungal plant pathogen *Verticillium dahliae* and relates the importance of epigenetic transcriptional regulation and other nuclear processes more broadly in fungal plant pathogens.

## Introduction

Arguably, one of the most important transition towards eukaryotic evolution has been cell compartmentalization, which allowed physical separation of diverse cellular processes [1]. One of the organelles that arose from this compartmentalization is the nucleus, the organelle that harbors most of the DNA of the eukaryotic cell. Nuclear processes include those that are required for short-term cell response, such as transcription, as well as processes that ensure long-term survival and inheritance, such as DNA replication and DNA repair.

Regulation of DNA-templated processes involves the histone code, made up of posttranslational chemical modifications to DNA-interacting histone proteins that help regulate genome functionality [2,3]. Eukaryotes have 4 canonical histone proteins (histone 2A, histone 2B, histone 3, and histone 4) that form globular octameric protein complexes by incorporation of 2 monomers of each histone protein [4]. By binding 145–147 bp of DNA, these protein complexes form nucleosomes that provide the packaging that is required to fit DNA into the confined space of the nucleus [4,5]. Each histone protein carries a flexible N-terminal tail that extends away from the nucleosome complex. These histone tails are enriched for amino acid residues that can undergo chemical modification, such as methylation, acetylation, and phosphorylation [6]. In addition to modifications to canonical histones, eukaryotes can also incorporate histone variants that substitute the core canonical histones in their nucleosomes [7,8]. The combination of histone modifications and variants cannot only locally regulate nuclear processes, but also more globally. Locally, nuclear processes can be regulated through histone modifications or variants that serve as recognition sites for enzymes that act on DNA [2,9–11], whereas they can affect DNA-accessibility over larger chromosomal regions and shape three-dimensional (3D) genome arrangements within the nucleus, leading global effects [12,13].

**Fig 1:**
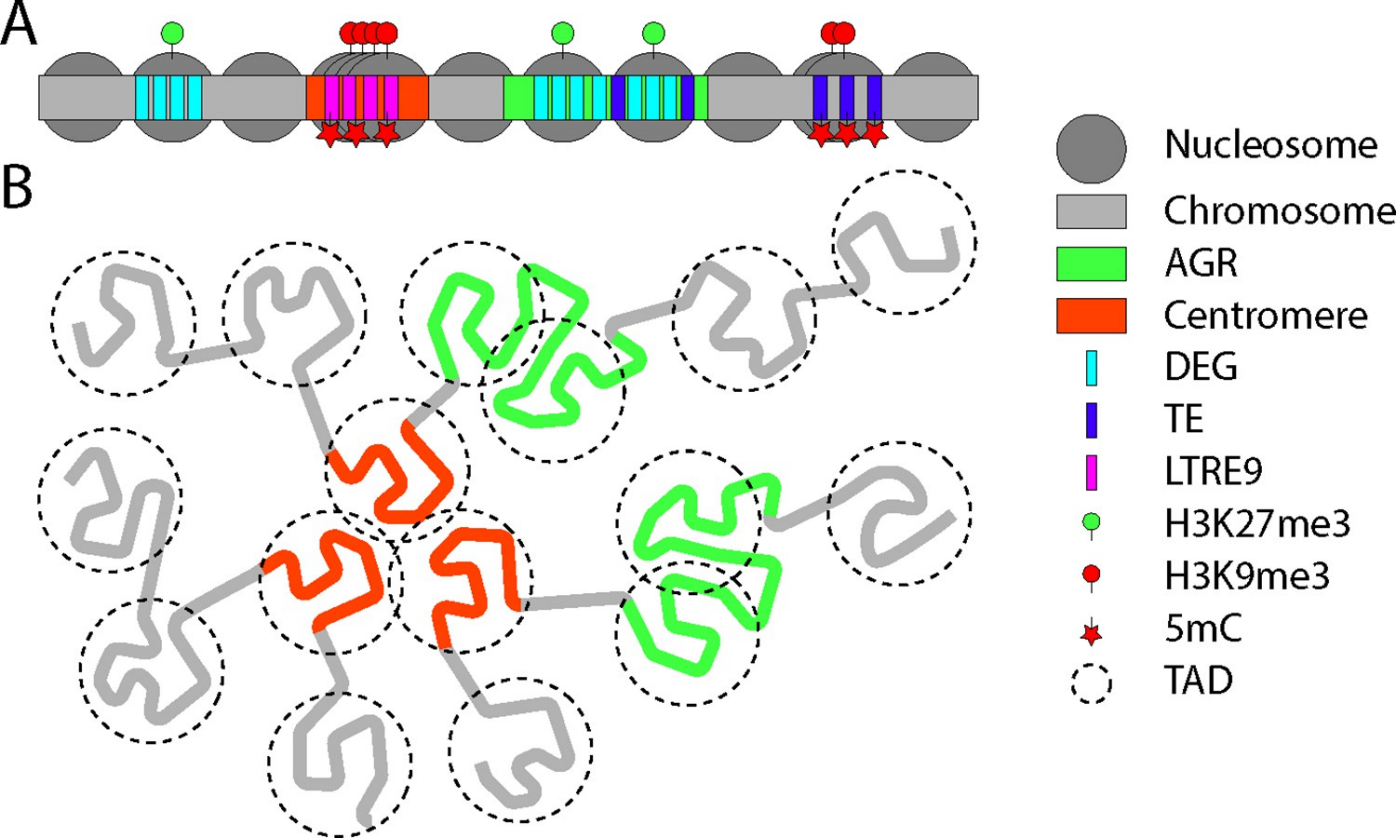
A model of genome organization and epigenetic modifications in *V*. *dahliae*. Chromosomal regions differentially display distinct chromatin features, associated with differences in 3D genome organization. (A) Representation of the chromatin structure on a linear *V*. *dahliae* chromosome. AGRs (green blocks) display a distinct open (uncondensed) chromatin profile, in which the nucleosomes are marked by tri-methylation of histone 3 lysine 27 (H3K27me3, green circles) [27]. H3K27me3-marked regions, consisting of AGRs, as well as particular regions of the core genome, are enriched for differentially expressed genes in vitro and in planta (DEGs, blue blocks) [35]. Centromeres (orange blocks) in *V*. *dahliae*, but not in all sister species are specifically associated with the LTR retrotransposon LTRE9 (pink blocks) [37]. The chromatin profile at centromeres consists of tightly packed nucleosomes that are marked by tri-methylation of histone 3 lysine 9 (H3K9me3, red circles) and by DNA-methylation (5mC, red stars) [36,37]. Besides LTRE9 TEs at the centromere, additional inactive TEs in the core genome are marked by H3K9me3 and 5mC, while active TEs in the AGRs are not associated with these marks. (B) Schematic 3D representation of the organization of 3 chromosomes in *V*. *dahliae*. Locally, genomic regions form TADs (indicated by dotted circles) that interact more strongly within the domain than with other domains. Intriguingly, TADs within AGRs are less well insulated and interact more freely with neighboring TADs [38]. Centromeres often form single TADs and display strong inter-centromeric interactions [37]. AGR, adaptive genomic region; TAD, topologically associating domain; TE, transposable element.

In filamentous fungi, the histone code was initially studied in the saprophyte *Neurospora crassa* [14,15]. In this species, histone modifications affect transcription, DNA replication, DNA repair, and chromosome segregation [14,16–21]. The impact of histone modifications have been studied in other filamentous fungi, including saprophytes as well as human pathogens, such as *Aspergillus* species [22,23], and in various plant pathogens [24–28]. One such plant pathogenic fungus is *Verticillium dahliae*, which causes vascular wilt disease in hundreds of host plants [29]. *V*. *dahliae* is an ascomycete, haploid fungus that reproduces predominantly asexually [30]. Genomic analyses on various strains uncovered that *V*. *dahliae* harbors a genome that evolved through large-scale chromosomal rearrangements, including duplications that have been followed by reciprocal gene losses [30–34]. Eventually, these processes resulted in a genome structure that can be characterized by core genomic regions that are shared by all strains and so-called adaptive genomic regions (AGRs, previously called “lineage-specific,” LS) that show a considerable degree of plasticity among strains [27,31]. Recently, we explored epigenetic features in relation to transcriptional regulation and genome evolution in *V*. *dahliae*, resulting in novel insights into the role of epigenetic modifications (Fig 1) [27,35–38]. In this review, we discuss epigenetic regulation of nuclear processes in plant pathogenic fungi, based on our recent findings for *V*. *dahliae*.

## Epigenetic mechanisms affect transcription

To colonize their plant hosts, plant pathogens secrete so-called effector molecules to support host colonization, often through tampering with host immunity [39,40]. The coordinated expression of effectors requires specific transcriptional regulation, likely balancing the need to subvert the host immune system with the potential cost of triggering immunity or other negative consequences of transcription [41,42]. Regulation of transcription in eukaryotes involves binding of transcription factors to promoter regions, followed by recruitment of DNA-dependent RNA polymerases that generate mRNA molecules [43]. In plant pathogenic fungi, only a few transcriptional regulators have been identified that are involved in effector gene expression. For instance, the transcriptional regulator Sge1 of *Fusarium oxysporum* f. sp. *lycopersici* is required for expression of particular proteinaceous effector genes during infection, and deletion of *Sge1* compromises pathogenicity on tomato [44]. Homologs of Sge1 were shown to also control expression of pathogenicity-related genes in several other plant pathogens [45–48]. Similarly, the transcription factor Pf2 regulates expression of some pathogenicity-related genes in various necrotrophic fungal plant pathogens [49–51]. The *Magnaporthe oryzae* regulator of G-protein signaling RGS1 acts as a transcriptional regulator by repressing the expression of numerous effector genes before plant penetration [52]. In these examples, the transcriptional regulators globally coordinate effector gene expression upon infection; however, it is important to note that individual effector genes are often expressed during specific stages of infection [41,42,53]. Therefore, additional transcriptional regulatory mechanisms are required to accurately express effector genes during infection.

The binding and recruitment of transcriptional machinery is influenced by chromatin condensation over broad genomic regions [10,54] and more locally by particular histone modifications that recruit or inhibit local protein binding [55,56]. Thus, epigenetic mechanisms are involved in the regulation of transcription. The chromatin at actively expressed genes is often hyperacetylated, while silent chromatin is hypoacetylated [57]. Therefore, histone acetylation may regulate transcription through activity of histone acetyl transferases (HATs) and histone deacetylase complexes (HDACs) [58,59]. Various fungal plant pathogens utilize HATs during infectious life stages [60–62], including the corn smut fungus *Ustilago maydis*, the banana pathogen *Fusarium oxysporum* f. sp. *cubense*, and the wheat pathogen *Fusarium graminearum*. In these fungi, deletion of genes encoding HAT family members affected fungal virulence as well as lifestyle switches [60–62]. In *U*. *maydis*, activity of the HDAC member Sir2 is involved in pathogenic development, although it is unclear whether Sir2 is truly active in deacetylation [63]. Recent results in *Saccharomyces cerevisiae* have raised concerns about the role of histone acetylation in transcriptional regulation, as temporal experiments show that transcriptional activation occurs before histone acetylation, which suggests that even though histone acetylation may be involved in transcriptional regulation during disease development, it may not directly activate transcription [64].

**Fig 2:**
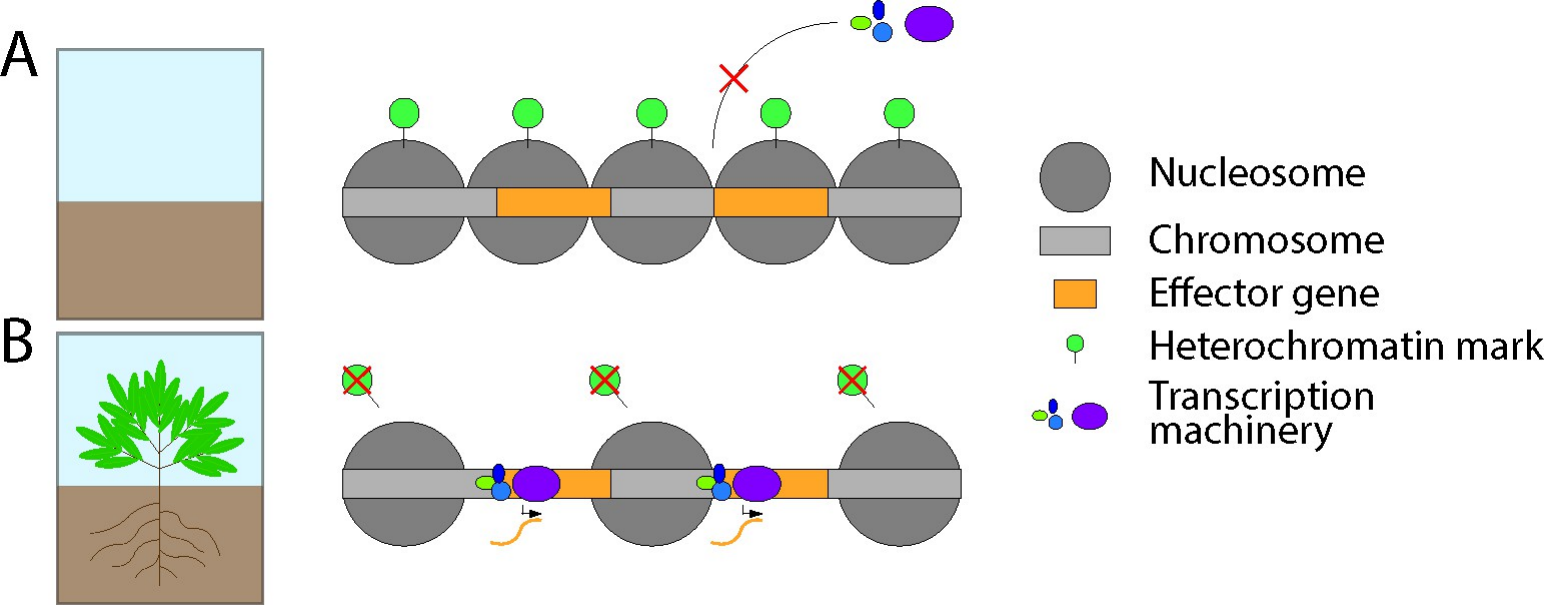
Hypothesis on epigenetic regulation of effector gene expression in fungal plant pathogens. (A) Chromosomal regions containing effector genes (yellow blocks) are marked by heterochromatin-associated histone modifications (green circles) and the chromatin is condensed and inaccessible to transcription machinery (colored ellipses) when the pathogen does not require effector gene expression. (B) Upon plant recognition, chromosome regions containing effector genes lose their heterochromatin-associated histone modifications and the chromatin decondenses and becomes accessible to the transcription machinery.

During axenic cultivation of numerous plant pathogenic fungi, when effector genes are generally repressed, DNA regions coding effector genes were enriched for H3K9me3 and H3K27me3, and mutants in genes encoding the histone lysine methyltransferases KMT1 and KMT6, respectively, that are responsible for the deposition of these marks displayed de-repressed effector gene expression [24–26,28,65–68]. As the histone modifications H3K9me3 and H3K27me3 are generally associated with inaccessible heterochromatin, these findings led to the hypothesis that genomic regions containing effector genes are heterochromatic, and therefore, inaccessible to the transcriptional machinery, when the pathogen grows outside of the host plant (Fig 2). Consequently, in order to express the effector genes that facilitate infection, pathogens are generally hypothesized to require chromatin de-condensation at effector gene-containing regions, possibly through depletion of H3K9me3 and H3K27me3 (Fig 2) [25,69,70].

To study epigenetic regulation of effector gene expression, analyses are preferably performed in planta, the niche where fungal plant pathogens naturally occur and the context in which they are most studied. However, epigenetic studies of fungal chromatin during plant infection are typically impeded by the usually low pathogen-to-plant biomass, resulting in excessive amounts of plant-derived sequences from interaction samples [69]. Consequently, to our knowledge, no analyses on genome-wide presence of histone modification dynamics during infection have yet been reported. Current reports that discuss epigenetic regulation of transcription in planta rely on association with H3K27me3 in vitro, genetic perturbation altering global H3K27me3 deposition, or the analysis of only a few representative genes in planta [25,28,65,67,68]. Therefore, the full scope of epigenetic regulation and histone modification dynamics that occur during fungal-host infection remains unknown.

In agreement with the notion that effectors are heterochromatically silenced in vitro, *V*. *dahliae* genomic regions harboring effector genes and other environmentally regulated genes are enriched for H3K27me3 in vitro, and loss of H3K27me3 in the *Set7* deletion mutant, leads to transcription of previously marked genes [27,35]. These results are consistent with the idea that transcriptional regulation of effector gene expression in *V*. *dahliae* may also occur in a similar fashion as proposed in other filamentous plant pathogens [27,35]. However, experiments to test the link between histone modification status and transcriptional activity between different axenic conditions reveal a different situation [35]. Here, we find that although H3K27me3 is enriched at effector loci, and it is required for repression, our results do not suggest that depletion of H3K27me3 is the major event leading to transcriptional activation [35]. This is because differential expression of genes located in H3K27me3-marked domains do not require concomitant changes in H3K27me3 status [35], although we cannot fully exclude that H3K27me3 is lost in only a subset of fungal nuclei during effector gene activation and that chromatin immunoprecipitation followed by high-throughput sequencing (ChIP-seq), which is used to determine the presence of this marks, does not have sufficient sensitivity to detect such small differences within the population. Loss of H3K27me3 in the plant pathogenic fungi *Fusarium graminearum*, *Fusarium fujikuroi*, and *M*. *oryzae* similarly leads to induction of only a subset of previously H3K27me3-associated genes [24,26,65], showing that other factors are involved in transcriptional regulation as well. Interestingly, the lack of concomitant removal of H3K27me3 and transcriptional activation may have to do with the finding in *V*. *dahliae* that H3K27me3-marked chromatin is not nearly as condensed as H3K9me3-marked regions during fungal cultivation in vitro (Fig 1A) [27]. Thus, our results in *V*. *dahliae*, combined with the results in other fungi, indicate that the previously postulated hypothesis does not fully describe the role of H3K27me3 in regulation of effector gene expression. Rather, H3K27me3 presence may serve to help repress transcription during unwarranted conditions and serve as a binding site or nucleation point to regulate transcriptional activation upon detection of particular environmental signals.

Proteins recognizing and binding to histone modifications are generally known as histone readers and can function in various cellular processes, including transcriptional regulation [71]. For instance, the TAF3 subunit of the basal transcription complex TFIID specifically binds H3K4me3 in a human cell line [72]. In response to genotoxic stress, TAF3 is recruited to H3K4me3-marked genes to stimulate formation of the preinitiation complex and thereby regulates initiation of gene expression [73]. In contrast, binding of H3K4me3 in another human cell line by the histone reader ING2, a subunit of the mSin3a histone deacetylase complex, leads to rapid repression of gene expression [74]. It is also possible for a single histone reader to recognize multiple histone marks and elicit different transcriptional activity depending on the other present histone marks. For instance, the *Arabidopsis* histone reader EARLY BOLTING IN SHORT DAY (EBS) recognizes both H3K4me3 and H3K27me3 and can switch between binding those histone marks to balance gene expression [75]. As the histone code in plant pathogens has only been partially scrutinized, it is possible that H3K4me3 or another, perhaps yet unidentified, histone modification works in conjunction with H3K27me3 to regulate transcription of effector genes. Although cooperation between histone marks as target of a histone reader is unknown in fungi, it is clear that histone marks themselves do affect each other’s deposition. An interesting example of such interaction between histone modifications occurs between H3K36me3 and H3K27me3 [20,76]. H3K36me3 deposition by the *F*. *fujikuroi* histone methyltransferase Ash1 occurs predominantly in the transcriptionally inactive subtelomeric regions, and this presence of H3K36me3 antagonizes deposition of H3K27me3 [76]. In contrast, experimentally induced H3K27me2/3 deposition in *N*. *crassa* prevalently occurred at Ash1-deposited H3K36me3 domains [20]. In both fungi, H3K36me3 deposited by another histone methyltransferase, Set2, occurs at transcriptionally active genes [20,76]. These examples indicate that the genomic context, and the accompanying epigenetic context, matters for the functionality of histone marks, and that this may differ between fungal species. Thus, the relatively stable presence of H3K27me3 observed in *V*. *dahliae* may lead to different transcriptional outputs depending on the co-occurrence of other histone marks and on specific interactions with opposing histone readers. Interestingly, the recently described H3K27me3 histone readers, EPR-1 and BP1, contribute to transcriptional repression in the filamentous fungi *F*. *graminearum* and *N*. *crassa* [77,78]. As these histone readers inhibit binding of transcriptional machinery, it is conceivable that dynamic presence of such readers over stable H3K27me3 domains regulates transcription.

Besides potentially functioning as a direct regulator of transcription, H3K27me3 may also affect transcriptional regulation through shaping 3D organization of the genome within the nucleus. In other systems, including animals, plants, and fungi, genomes have been shown to display long-range intra-chromosomal and inter-chromosomal interactions [79–82]. At smaller genomic distances, 3D organization results in the formation of topologically associating domains (TADs), which are local genome regions that interact preferentially within themselves, but not with adjacent genomic regions [13,83]. The exact functionality of TADs is still under debate [84–87], but TADs have frequently been associated with transcriptional regulation, for instance, by facilitating enhancer–promoter interactions [83,88–90]. Connecting the epigenome to the 3D genome, H3K27me3 has been shown to affect both local and global 3D chromosome organization [91–93]. Thus, H3K27me3 may be involved in structuring the 3D genome to help regulate transcription, for instance, by providing conducive local 3D-chromatin micro-environments containing components of the transcriptional machinery. Our observations in *V*. *dahliae* indicate that genes within the H3K27me3-associated TADs in AGRs are more likely to be transcriptionally co-regulated than genes in core TADs [38]. This suggests that H3K27me3 may be involved in organizing the local 3D genome to help direct the divergent transcriptional profiles seen for TADs in AGRs compared to the core genome. The local 3D genome organization in *N*. *crassa* also differs between genomic regions, with transcriptionally silent regions displaying random internal contacts, while organization of active chromatin is more reminiscent of TAD structure [94]. However, this is mostly H3K27me2/3 independent, as H3K27me3 is not present on all the regions that display such random internal contacts [81,92,94]. Recently, such differences in 3D genome organization were investigated and discussed with respect to their impact on genome-wide DNA-templated processes [95].

## Epigenetic mechanisms affect genome evolution

To survive over evolutionary timescales as a species, organisms need to gain, lose, or alter proteins performing particular functions through genome evolution. This may be particularly relevant for plant pathogens, because plants recognize the presence of potential pathogens, or their activity, through intra- and extracellular immune receptors that bind non-self or modified-self ligands, collectively named invasion patterns [40]. Such invasion patterns can be structural components of pathogen cells, also known as microbe-associated molecular patterns (MAMPs), signatures of pathogen-induced plant damage, termed damage-associated molecular patterns (DAMPs), or proteins or metabolites produced by the pathogen during host invasion [40,96]. In order to circumvent recognition by the host plant, successful pathogens evolve to secrete novel proteins that inhibit recognition or evolve to lose or mutate the recognized molecule [40]. In turn, plants evolve novel immune receptors to again restrict pathogen dissemination, leading to a co-evolutionary arms-race between plants and their pathogens, where single gene loss, gain, or mutation can alter the outcome of the interaction from compatible to incompatible or vice versa [39,96,97].

Genetic variation occurs via a combination of mechanisms, including DNA replication errors, external mutagens, chromosomal crossover events, transposable element (TE) activity, partial or whole-genome duplications, chromosome gain or loss, large-scale chromosome rearrangements, etc. [98]. In some cases, the generated genome variation leads to increased cell or organism viability, potentially affecting their frequency in the population, ultimately leading to evolution. Even though genome evolution is considered a stochastic process, it was found that various plant pathogens harbor genomic regions that display increased frequencies of genetic variation, while the majority of the genome, which is typically designated as the core genome, remains evolutionary rather stable [33,99–106]. This compartmentalization can generally be described as regions that differ more frequently between strains of a species compared to regions that are more highly conserved, often termed the two-speed genome in pathogenomics [99,100]. The “fast-evolving,” plastic compartments of a two-speed genome are typically TE-rich [100], indicating that TE activity may be one of the more important drivers for evolution. The plastic compartments of *V*. *dahliae* are represented by the TE-rich AGRs that evolved through large-scale chromosome rearrangements and segmental duplications, followed by reciprocal gene losses [31,33]. Interestingly, the TEs located in AGRs are relatively young, transcriptionally active, and more frequently polymorphic when compared with TEs in the core genome, suggesting that these TEs actively contribute to shaping of the AGRs [33,34,106]. Additionally, the polymorphic TEs in AGRs are associated with in planta highly expressed pathogenicity-related genes, suggesting that TEs may be involved in transcriptional regulation as well [34,106].

Even though TE activity is beneficial to a certain extent, TE overactivity can be detrimental to genome stability, and, therefore, TEs are generally epigenetically silenced [107–110]. In fungi, genomic regions that are enriched for TEs are often epigenetically silenced by H3K9me3 and cytosine methylation (5-methylcytocine, 5mC) [111–115]. Similarly, in *V*. *dahliae* we found that H3K9me3 and 5mC co-localize on TE-rich genomic regions (Fig 1A) [27,36,37]. However, even though 5mC is generally thought to be involved in transcriptional silencing, we observed that loss of 5mC did not induce TE transcription, whereas loss of H3K9me3, and the accompanying loss of 5mC, leads to the induction of numerous TEs [36]. Thus, 5mC is not strictly necessary for TE silencing. Instead, 5mC may be subject to spontaneous deamination, causing C to T mutations, and thus potentially rendering affected TEs permanently inactive [116]. Cytosine deamination as a driver of genome evolution is well accepted in various taxonomic groups [117–119]. For instance, simulations of DNA sequence evolution indicated that mutational pressure by cytosine deamination was vital for the evolution of isochore structures in the mammalian genomes [118]. Additionally, cytosine deamination has been proposed to constitute one of the main evolutionary forces in generating new transcription factor-binding sites in the human genome [120]. However, as cytosine methylation is mainly restricted to genomic regions containing TEs in *V*. *dahliae*, spontaneous deamination is likely mainly involved in the inactivation of TEs and less so in genome evolution more broadly. It needs to be noted that there is no evidence for repeat-induced point mutation (RIP) in the presumed asexual fungus *V*. *dahliae*, which could alternatively cause C to T mutations instead of spontaneous deamination [27,31,33].

Previous studies in *V*. *dahliae* indicated that relatively young and active TEs are associated with the evolution of AGRs [27,33]. Interestingly, the TEs in these AGRs have a lower fraction of C to T mutations (represented by the composite repeat-induced point mutation index, CRI) and display lower association with H3K9me3 and 5mC [27,33,34]. This indicates that C to T mutations happen more frequently in TEs that are marked with 5mC, and thus that spontaneous deamination may be a true end result of DNA methylation, but also that a particular subset of TEs is devoid of H3K9me3 and 5mC. It is interesting to speculate that this contributes to TE activity and helps drive evolution within the AGRs, but further direct experimental evidence is needed.

It remains unclear what dictates the disparate TE silencing observed in *V*. *dahliae*, but also other fungi [121,122]. One explanation is simply that of natural selection, where fungal cells with active TEs in the core genome suffer fitness penalty and do not flourish, while cells with active TEs in the AGRs experience less detrimental effects, and thus survive more frequently. Alternatively, the presence of specific epigenetic features of AGRs may constrain the deposition of H3K9me3 and 5mC on TEs in the plastic genome, thereby permitting elevated TE activity within AGRs. Furthermore, it is very intriguing that pairwise clustering of AGRs in the 3D genome corresponds with segmental duplications underlying their evolution [38]. More generally, we observed in *V*. *dahliae* that TADs in the H3K27me3-associated AGRs are weaker insulated than TADs in the core genome, meaning that TADs in AGRs are less well separated from their neighboring TADs (Fig 1B) [38]. We speculate that this organization may allow more promiscuous interactions and may promote higher genome instability. It is unclear what makes that AGRs cluster in the 3D genome, nor what the function of these membrane-less nuclear sub-compartments, so-called nuclear bodies, may be [123–125]. Various types of nuclear bodies have been described, including nucleoli, Cajal bodies, nuclear speckles, nuclear stress bodies, and polycomb bodies, of which some are formed on chromatin and embed DNA, while others form in the nucleosol and do not contain DNA, but rather interact with chromatin [125,126]. Chromatin-containing nuclear bodies are proposed to form by a process called phase separation through the activity of self-aggregating chromatin-binding molecules or through the activity of individual chromatin bridging factors that cross-link separate chromatin sections, without self-aggregation [126]. Nuclear bodies formed with the non-aggregating bridging factors are usually less stable, as these molecules can more readily disperse into the nucleoplasm, whereas nuclear bodies formed with self-aggregating molecules are more stable, and can exist independent of chromatin [126]. Interestingly, the *Drosophila* H3K9me3-interacting protein HP1 was shown to aggregate in vitro and to nucleate into foci during early heterochromatin domain formation, suggesting that aggregation of HP1 may drive heterochromatin domain formation [127]. As such, H3K9me3-marked heterochromatin at distal genomic regions may cluster in the nucleus through the presence of HP1, for instance, at centromeres [128]. Similarly, H3K27me3-marked heterochromatin is bound by the polycomb repressive complex 1 (PRC1), of which the CBX2 protein member is capable of assembly through phase separation [129]. PRC1 components are absent from most fungi [130,131], yet other H3K27me3-readers may have an analogous function in fungi. Thus, it is possible that epigenetic differences between the AGR and core segments of *V*. *dahliae* [38] function to segregate core and AGR regions to promote the activity of TEs in AGRs in such a way that the core genome remains largely unaffected. In addition to histone modifications influencing TE activity, there is also mounting evidence that histone modifications impact the generation of DNA variation. Two recent studies used mutation accumulation experiments in fungi, subculturing the strains under minimal selection, allowing subsequent identification of de novo genome variation [132,133]. In the plant pathogen *Zymoseptoria tritici*, the causal agent of Septoria leaf blotch, heterochromatin defined by the presence of H3K9me3 and H3K27me3 impacted the accumulation of DNA variation. The genetic loss of H3K9me3 led to a significantly higher base mutation rate, as well as more frequent loss of specific accessory chromosomes, while genetic loss of H3K27me3 accounted for a small reduction in base accumulation in some genomic regions [132]. In the filamentous fungal saprophyte *Neurospora crassa*, mutation accumulation was found to be higher in both H3K27me3- and H3K9me3-marked regions [133]. Further research is needed to understand the mechanisms leading to increased variation accumulation rates, but the results clearly indicate that the epigenome is playing an important role in this nuclear process.

All organisms evolved DNA repair mechanisms that correct damage to the genome. Various DNA repair mechanisms exist, including double-strand break repair by homologous recombination or by nonhomologous end joining, and nucleotide and base-excision repair pathways [134,135]. Interestingly, the histone code has been implicated in these DNA repair mechanisms. For instance, histone methylation of lysine 79 on the tail of histone 3, and of lysine 20 on histone 4, as well as phosphorylation and ubiquitination of histone variant H2AX are involved in recruitment of double-strand break machinery [135]. Additionally, budding yeast mutants lacking the N-terminal tails of histones H2A and H3 displayed increased mutation rates due to deficient base-excision repair, indicating that chromatin plays an important role in this DNA repair mechanism [136]. Analysis of CRISPR-Cas–induced DNA double-strand breaks in *M*. *oryzae* indicated that multiple DNA repair pathways may function differently across the genome during DNA repair [137]. As such, the creation and repair of DNA lesions may be a significant driver of DNA variation fueling fungal pathogen evolution [138]. In fact, research in *V*. *dahliae* identified sequence signatures of double strand-break repair machinery at sites of chromosomal rearrangements, indicating that the evolution of the two-speed genome in *V*. *dahliae* involved erroneous double-strand break repair (Faino and colleagues). As these mechanisms are in part regulated on chromatin, there is increasing evidence that epigenetics is important for genome evolution [139].

## Epigenetic mechanisms affect cell division

For mitotic and meiotic replication, organisms first require DNA replication, followed by segregation of chromosome pairs, and finally cell division [140]. DNA replication starts with unwinding and separation of DNA strands, resulting in formation of replication forks in which DNA-polymerases attach to the DNA. The formation of replication forks is favored in genomic regions with hyperacetylated euchromatin [141,142], and the timing of replication is further regulated on chromatin [142–144]. Heterochromatic regions generally replicate later during the mitotic S-phase and this process involves histone acetylation and methylation, as well as the activity of histone readers recognizing heterochromatin-associated histones [145–147]. Interestingly, an exception to late replicating heterochromatic regions are the centromeres in the fungus *Schizosaccharomyces pombe*, which are heterochromatic yet were shown to replicate relatively early [148].

After DNA replication, the generated chromosome pairs segregate through formation of microtubule spindles that attach to centromeric regions present on each chromosome [149]. Fungal centromeres vary significantly in composition and size between species, ranging from point centromeres of approximately 125 bp in size to regional centromeres of a few kb up till a few hundreds of kb [150,151]. Even though fungal centromeres vary widely, their chromatin is always characterized by presence of nucleosomes carrying the histone H3 variant CenH3 [150,151]. CenH3 is essential for centromere function, as it is the chromatin component that connects chromosomes to the microtubule spindle via the proteinaceous kinetochore complex [149]. Besides CenH3, fungal centromeres are often epigenetically characterized by H3K9me3 and 5mC [151,152], which we also found to be present at *V*. *dahliae* centromeres (Fig 1A) [37]. Additionally, in various plants and animals, a large set of histone modifications have been associated with centromeres [153]. The conservation of epigenetic profiles at centromeres in different organisms indicates that the epigenetic landscape likely plays a crucial role in centromere function, and thus in cell division. Such role of the epigenetic landscape, and perhaps especially of H3K9me3, may entail the formation of a nuclear sub-compartment and thereby drive the physical clustering of centromeres. Interestingly, incorporation of the *S*. *pombe* CenH3 homolog CENP-A is promoted by nearby heterochromatin, and heterochromatin-bearing minichromosomes in *S*. *pombe* localize close to centromeres, suggesting that heterochromatin formation drives the nuclear positioning of centromeres [154]. The crucial role of the epigenetic landscape in centromere functioning is further supported by studies into neocentromere formation, occurring upon centromere defects, showing that neocentromeres often form in H3K9me3-marked genomic regions [155]. However, neocentromeres in the human pathogenic fungus *Cryptococcus deuterogattii* are formed in genic regions that are not associated with H3K9me3 and 5mC [156,157], suggesting that these heterochromatic features are not essential. Moreover, *C*. *deuterogattii* chromosomes lacking their original centromere are unstable and undergo chromosomal fusions, after which the neocentromere loses its function [156,157]. These results suggest that although H3K9me3 and 5mC may not be essential for centromere function and cell viability in short-term, they are important for genome stability over longer evolutionary timescales.

## Concluding remarks

In this review, we have highlighted the importance of epigenetics in the regulation of nuclear processes in fungal plant pathogens. Moreover, we describe experimental evidence that genomic regions containing effector genes are characterized by the presence of heterochromatic features, but it remains to be seen if in planta transcriptional activation requires chromatin de-condensation or epigenome remodeling. As we show that differential gene expression in vitro for only a subset of genes located in H3K27me3 domains involves local H3K27me3 depletion, we postulate that gene expression in planta may display local H3K27me3 depletion, but that it is not strictly required. To demonstrate this, future studies will require a functional and reliable procedure to perform in planta chromatin immunoprecipitation assays or other types of nuclear capture or single-cell interrogation.

Histone proteins are not only found in eukaryotes, but are also common in archaea [158,159], indicating that the potential for epigenetic regulation using histones is evolutionary ancient. As such, it is not surprising that nuclear processes have evolved to heavily rely on epigenetic regulation. This evolution resulted in an intricate mechanism, the histone code, in which particular histone modifications may have multiple different functions depending on their genetic localization and the co-occurrence with additional modifications. Therefore, it will be difficult to predict how the function of the genome is affected by specific changes in the histone code. Advances in single-cell sequencing and epigenome analyses [160,161] will deepen the understanding of epigenetics by providing increasingly more fine-grained information about the regulation and output of the epigenome. While the major focus of epigenetic research has been on transcriptional regulation, there is substantial evidence that the epigenome impacts the evolution of genomes, including that of fungal pathogens. This will be an important area to develop a mechanistic understanding in order to predict and intervene on the development of emergent fungal pathogens of plants and animals.
